# Laparoscopic surgery does not reduce the need for red blood cell transfusion after resection for colorectal tumour: a propensity score match study on 728 patients

**DOI:** 10.1186/s12893-022-01569-0

**Published:** 2022-03-31

**Authors:** Giulia Turri, Giovanni Malerba, Gabriele Gecchele, Cristian Conti, Federica Randon, Pierluigi Piccoli, Giorgio Gandini, Domenico Girelli, Alfredo Guglielmi, Corrado Pedrazzani

**Affiliations:** 1 Department of Surgical Sciences, Dentistry, Gynecology and Pediatrics, Unit of General and Hepatobiliary Surgery, University of Verona, Verona, Italy; 2 Department of Neurosciences, Biomedicine and Movement, University of Verona, Verona, Italy; 3 Department of Transfusion Medicine, Verona University Hospital, Verona, Italy; 4 Department of Medicine, Section of Internal Medicine, University of Verona, Verona, Italy; 5 U.O.C. di Chirurgia Generale e Epatobiliare, Policlinico “G.B. Rossi”, Piazzale “L. Scuro” 10, 37134 Verona, Italy

**Keywords:** Colorectal surgery, Laparoscopy, Blood loss, Red blood cells transfusion

## Abstract

**Background:**

Patients with colorectal tumour often present with anaemia, and up to 60% will receive red blood cells (RBC) transfusion. Some evidence suggests a correlation between RBC transfusion and worse outcomes. Since laparoscopy minimizes intraoperative blood loss, we retrospectively investigated its role in reducing haemoglobin (Hb) drop and requirements for postoperative RBC transfusions.

**Methods:**

Patients were identified from consecutive cases undergone elective surgery for non-metastatic colorectal tumour between 2005 and 2019. Laparoscopic cases were matched 1:1 with open controls through propensity score matching (PSM). The main outcome measures were postoperative Hb drop and requirement for RBC. The secondary aim was evaluation of risk factors for postoperative RBC transfusions.

**Results:**

After application of PSM, 364 patients treated by laparoscopy were matched with 364 patients undergone open surgery. The two groups presented similar clinical and pathological characteristics, as well as comparable postoperative outcomes. 56 patients in the open group and 47 in the laparoscopic group required postoperative RBC (*P* = 0.395). No difference was observed in terms of mean number of RBC units (*P* = 0.608) or Hb drop (*P* = 0.129). Logistic regression analysis identified preoperative anaemia and occurrence of postoperative complications as relevant risk factors for postoperative RBC transfusion, while surgical approach did not prove statistically significant.

**Conclusion:**

Laparoscopy did not influence postoperative requirements for RBC transfusions after elective colorectal surgery. Preoperative anaemia and occurrence of postoperative complications represent the major determinants for postoperative transfusions after open as well as laparoscopic surgery.

**Supplementary Information:**

The online version contains supplementary material available at 10.1186/s12893-022-01569-0.

## Introduction

Lower tract gastrointestinal bleeding and anaemia are frequent events in newly diagnosed colorectal cancer (CRC) [1, 2]. Though attention has been paid to blood management in recent years, the number of red blood cell (RBC) transfusions after CRC surgery remains notably high [3–5], ranging from 5 to 60% [6–8].

RBC transfusions have been reported to impair short- as well as long-term outcomes after cancer surgery. Specifically, in the context of CRC surgery, two large meta‐analyses, as well as other literature, suggested that perioperative blood transfusions may be associated with increased postoperative infection rates, increased length of hospital stay, increased mortality, higher cancer recurrence rates, and increased costs [6, 9–11].

Since its introduction, laparoscopy has demonstrated to minimize intraoperative blood loss, with randomized controlled trials comparing laparoscopic and open CRC resections quantifying the difference in 70 to 200 mL [12–16]. Though the difference in intraoperative blood loss is demonstrated, little data exist on the real benefit of one approach over the other in terms of haemoglobin (Hb) level drop and need for postoperative blood transfusions [17, 18].

The primary purpose of this retrospective propensity score matching study was to evaluate the role of laparoscopy in reducing the postoperative drop in Hb levels and the requirement of RBC transfusions compared to open surgery after elective CRC resection. The secondary aim was to investigate the risk factors for postoperative RBC transfusions.

## Methods

### Inclusion criteria and population under study

**Fig. 1:**
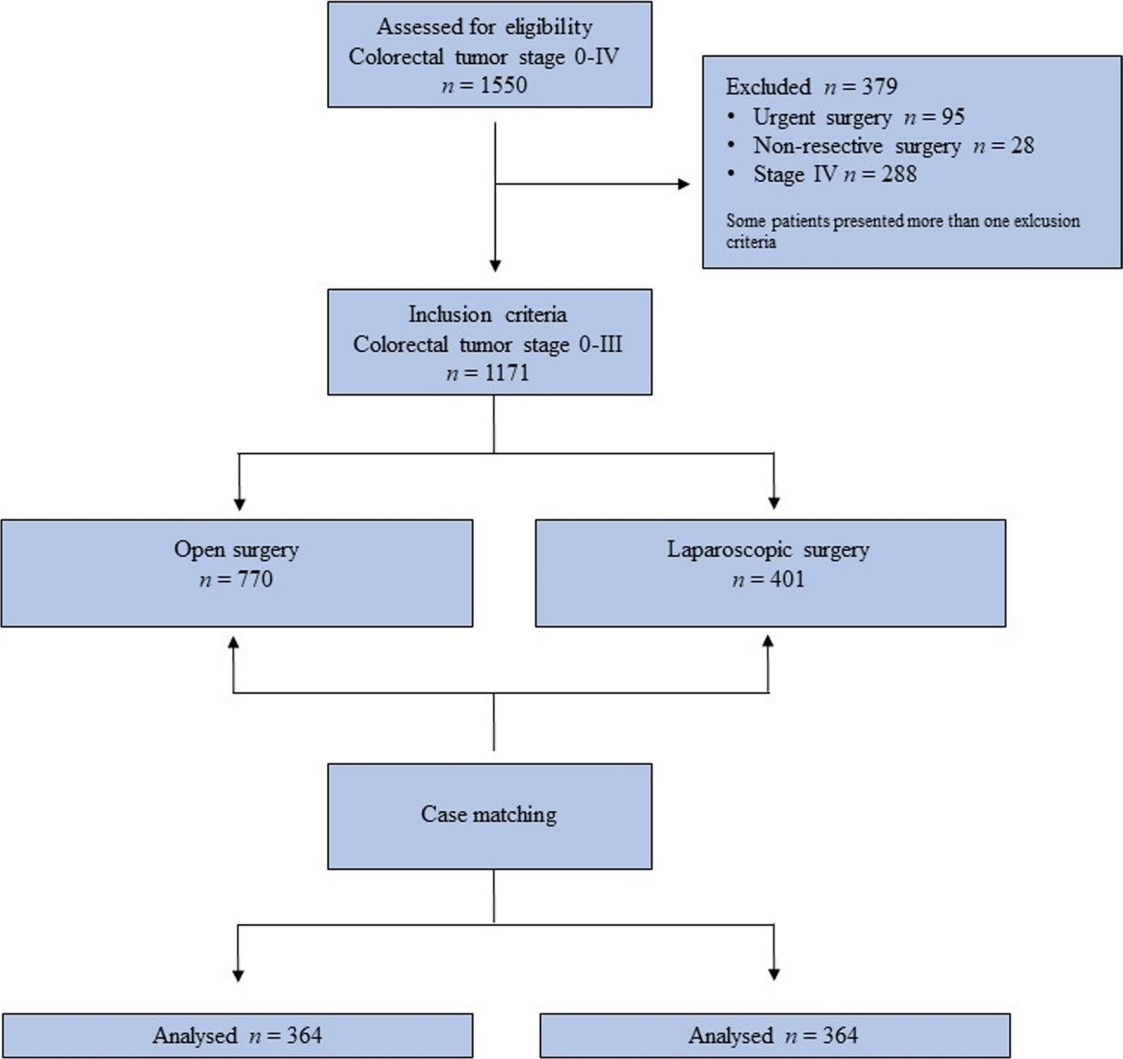
Diagram showing the process of patients’ selection and inclusion

The original patient population consisted of all patients undergoing surgery for colorectal tumour (1550 cases) at the Division of General and Hepatobiliary Surgery, University of Verona Hospital, between January 2005 and June 2019. Inclusion criteria were age of 18 years or older, elective laparoscopic and open resection, histology-proven colorectal tumour or adenomas not amenable of endoscopic resection, absence of metastases and minimum follow-up of 90 days. Patients with other colonic malignancies (e.g., neuroendocrine tumors, lymphomas) were not included. Figure 1 shows the process of patients’ selection and inclusion into the study. A total of 1171 patients fulfilled the inclusion criteria and entered the study, of which 401 (34.2%) underwent laparoscopic surgery. After application of propensity score matching (PSM) technique, the study group consisted of 364 patients undergone laparoscopic resection, who were matched 1:1 to 364 patients undergone open surgery within the study period.

### Preoperative work-up and histopathological staging

Prior to surgery, all patients were staged with standard blood exams, colonoscopy, chest–abdomen–pelvis computed tomography and measurement of carcinoembryonic antigen. Additional imaging studies including magnetic resonance or transrectal ultrasound were used for staging of rectal cancer. Liver magnetic resonance and positron emission tomography were considered in case of uncertain lesions to rule out the presence of metastatic disease. Pathology specimens were reported according to the 7th Edition of the American Joint Committee on Cancer (AJCC) and the Union International Contre Le Cancer (UICC) criteria [19].

### Extent of surgery and postoperative course

The complete excision of the tumour (R0 resection) was the main goal of surgery. The extent of surgery was planned according to patient condition, tumour location and stage. The choice of the surgical approach was based on surgeon’s preference. Both in open and laparoscopic surgery, anatomical resections with ligation of the vessels at their origin were preferred in order to harvest an adequate number of lymph nodes [20, 21]. Extent of surgical resection was never influenced by the adoption of laparoscopic surgery. Data on estimated intraoperative blood loss (EBL) were extracted from operative records. Postoperative complications were assessed according to the Clavien–Dindo classification, and graded as mild (grade I and II) or severe (grade III, IV, and V) [22].

### Assessment of haemoglobin levels and transfusion history

Preoperative Hb levels were acquired within 2 weeks from the date of surgery, and the nearest value to the date of operation was considered for Hb drop calculation. Postoperative Hb levels were checked on postoperative days (POD) 0, 1, 2 and 3 in all patients, and depending on clinical need thereafter. The greatest difference between preoperative and postoperative Hb levels was considered as Hb drop. Data regarding drop in Hb levels were gathered by reviewing the Medical Laboratory computer software system into which all laboratory results were entered.

Data regarding the number of RBC units transfused along with the date of transfusion were obtained starting from 30 days before (POD − 30) to 90 days (POD 90) after surgery by reviewing the Blood Transfusion Service database into which all information regarding blood transfusions are recorded. The need for RBC transfusions was determined on a case-by-case basis evaluating clinical course and Hb levels. Generally, Hb level lower than 8 g/dL was considered as a cut-off value in low-risk patients, whilst a value lower than 10 g/dL was considered for high-risk patients (i.e., patients who had a previous MI). No patients in the cohort developed severe anaemia with haemodynamic instability during the postoperative course.

Preoperative anaemia (suboptimal Hb level) was defined by a Hb level of less than 13 g/dL, for both sexes [23]. According to Hb levels on presentation, preoperative anaemia was subclassified as mild (Hb 11–12.9 g/dL) or moderate (Hb 8–10.9 g/dL) [24]. None of the patients underwent surgery with an Hb level lower than 8 g/dL.

### Data collection and statistical analysis

All clinical and pathological data were retrospectively collected and stored in a digital database. Demographic, clinical, surgical and pathology variables were analysed. Open and laparoscopic cases were paired using the propensity score matching (PSM) technique with the aim of obtaining two homogeneous groups. Multivariate logistic regression generated propensity scores, and open and laparoscopic cases were matched 1:1 considering age, gender, UICC-R category, TNM stage according to AJCC 7th Edition, need for preoperative RBC transfusions and preoperative Hb levels as covariates. The nearest neighbour method was used. After PSM was performed, differences between the two groups were assessed. Absolute standardized mean differences were estimated to evaluate post-match imbalance, and a standardized mean difference < 0.15 was considered a negligible difference in the mean or prevalence of a covariate between treatment groups. Additional file 2: Table S1 reports standardized mean differences for matching variables before and after matching.

Continuous data were reported as mean (+ standard deviation, SD) or median (interquartile range, IQR) while categorical data were reported as numbers and percentages. Comparisons between groups were made by Student’s t test or Mann–Whitney U test for continuous variables and Chi-squared test or Fisher’s exact test for categorical variables as appropriate. All statistical tests were two-sided and association were considered statistically significant at a nominal level of 0.05 (*P* < 0.05). Logistic regression models were used to estimate the strength of association between postoperative blood transfusions and other factors when including into the model relevant covariates. The analysis included the following variables: age (< median versus ≥ median), gender (male versus female), tumour location (right colon versus left colon versus rectum), American Society of Anaesthesiologists status (ASA 1–2 versus ASA ≥ 3), presence of preoperative anaemia (no anaemia versus mild anaemia versus moderate anaemia), preoperative RBC transfusions (no versus yes), depth of tumour invasion (< pT3 versus ≥ pT3), presence of nodal metastases (N+ versus N0), postoperative complications (no versus yes), severe postoperative complications (no versus yes), surgical complications (no versus yes), anastomotic leakage (no versus yes), bleeding (no versus yes), infective complications (no versus yes), surgical approach (laparoscopic versus open surgery). A screened *P*-value of < 0.10 at univariate analysis was considered for entering the covariate in the multivariate model after validating the absence of multicollinearity. Consequently, postoperative complications, severe postoperative complications, surgical complications, anastomotic leakage, bleeding, and infection complications were included in the model separately since their collinearity. Strength of the associations were reported as odds ratios with 95% confidence intervals (c.i.). Statistical analysis was performed using SPSS, version 23 (SPSS, IBM Corp., Armonk, NY, USA), and R, version 3.6.2, and the R packages “MatchIt” version 3.2 and “foreign” version 0.8–7.

## Results

### Cohort under study

Table 1 reports demographics and clinical-pathological characteristics for laparoscopic and open cases. Apart from a higher BMI in the open resection group (26.0 ± 4.1 versus 25.2 ± 3.6; *P* = 0.036), no significant differences were noted for other variables. In particular, the two groups were homogeneous in terms of tumour location, staging, and preoperative RBC transfusion.

**Table 1:** Demographic and clinical-pathological characteristics for the 728 under study according to treatment group

	Open resection *n* = 364	Laparoscopic resection *n* = 364	*P*
Age, years, mean ± SD	66.9 ± 10.6	66.5 ± 10.8	0.566
Male gender	211 (58)	196 (53.8)	0.269
Tumour location			0.354
Right colon	138 (37.9)	134 (36.8)	
Left colon	104 (28.6)	121 (33.3)	
Rectum	122 (33.5)	109 (29.9)	
BMI, kg/m^2^, mean ± SD	26.0 ± 4.1	25.2 ± 3.6	0.036
ASA ≥ 3	86 (23.7)	90 (24.7)	1
Preoperative Hb level, g/dL, mean ± SD	13.3 ± 1.8	13.3 ± 1.8	0.812
Presence of preoperative anaemia			0.796
None	221 (60.7)	228 (62.6)	
Mild	99 (27.2)	91 (25)	
Moderate	44 (12.1)	45 (12.4)	
Preoperative RBC transfusions			0.962
None	350 (96.2)	349 (95.9)	
One–two	6 (1.6)	7 (1.9)	
Three or more	8 (2.2)	8 (2.2)	
UICC-R status			1
R0	356 (97.8)	356 (97.8)	
R1	6 (1.6)	6 (1.6)	
R2	2 (0.5)	2 (0.5)	
Depth of tumour invasion			0.110
≤ pT2^a^	194 (53.3)	199 (54.7)	
pT3	109 (29.8)	123 (33.8)	
pT4	61 (16.7)	42 (11.5)	
Nodal involvement			0.914
pN0	203 (67.7)	215 (70.0)	
pN1	73 (24.3)	69 (22.5)	
pN2	24 (8.0)	23 (7.5)	
AJCC TNM stage			0.926
Stage 0–I^a^	171 (46.9)	180 (49.4)	
Stage II	95 (26.1)	92 (25.3)	
Stage III	98 (26.9)	92 (25.2)	

Numbers in parentheses are percentages unless specified otherwise

*SD* standard deviation

^a^Including 106 dysplastic adenomas (54 patients in open group and 52 patients in laparoscopic group)

Operative data for the two groups are shown in Table 2. A higher number of excised nodes (21.4 ± 1.8 versus 19.8 ± 11; *P* = 0.049) and a reduced length of hospital stay (7 [5–9] versus 9 [6–11]; *P* < 0.001) characterized laparoscopic resections. No other differences in operative data were observed. Specifically, comparable rates in postoperative mortality (*P* = 0.187), global complications (*P* = 0.587), complications graded as Clavien–Dindo equal or higher than 3 (*P* = 0.476) and need for reiterative surgery (*P* = 0.590) were recorded.

**Table 2:** Operative data for the 728 patients under study according to treatment group

	Open resection *n* = 364	Laparoscopic resection *n* = 364	*P*
Conversion	–	36 (9.9)	
Extent of surgery			0.433
Right hemicolectomy	136 (37.4)	133 (36.5)	
Left hemicolectomy	84 (23.1)	101 (27.7)	
Rectal resection	123 (33.8)	104 (28.7)	
Abdominoperineal excision	13 (3.5)	15 (4.1)	
Others	8 (2.2)	11 (3)	
No. of excised nodes, mean ± SD	19.8 ± 11	21.4 ± 10.8	0.049
Stoma formation			0.527
Ileostomy	64 (17.6)	51 (14)	
Colostomy	15 (4.1)	19 (5.2)	
30-day postoperative morbidity	132 (36.3)	124 (34.1)	0.587
Clavien–Dindo ≥ III	25 (6.9)	24 (6.6)	0.476
Anastomotic leakage	15 (4.1)	17 (4.7)	0.603
Abdominal bleeding	7 (1.9)	3 (0.8)	
Gastrointestinal bleeding	7 (1.9)	7 (1.9)	
Length of hospital stay, days, median (IQR)	9 (6–11)	7 (5–9)	< 0.001
Reiterative surgery	14 (3.9)	18 (4.9)	0.589
Unplanned readmission	–	5 (1.4)	0.590
30-day postoperative mortality	4 (1.1)	1 (0.3)	0.187

Numbers in parentheses are percentages unless specified otherwise

*SD* standard deviation; *IQR* interquartile range

Median (range) EBL for the laparoscopic and open cases was 40 (10–600) mL and 100 (50–450) respectively (*P* = 0.083), with only 15 cases (4.1%) in the laparoscopic group and 32 (8.7%) in the open group having an intraoperative blood loss greater than 200 mL.

### Transfusion history and hemoglobin levels variation

**Fig. 2:**
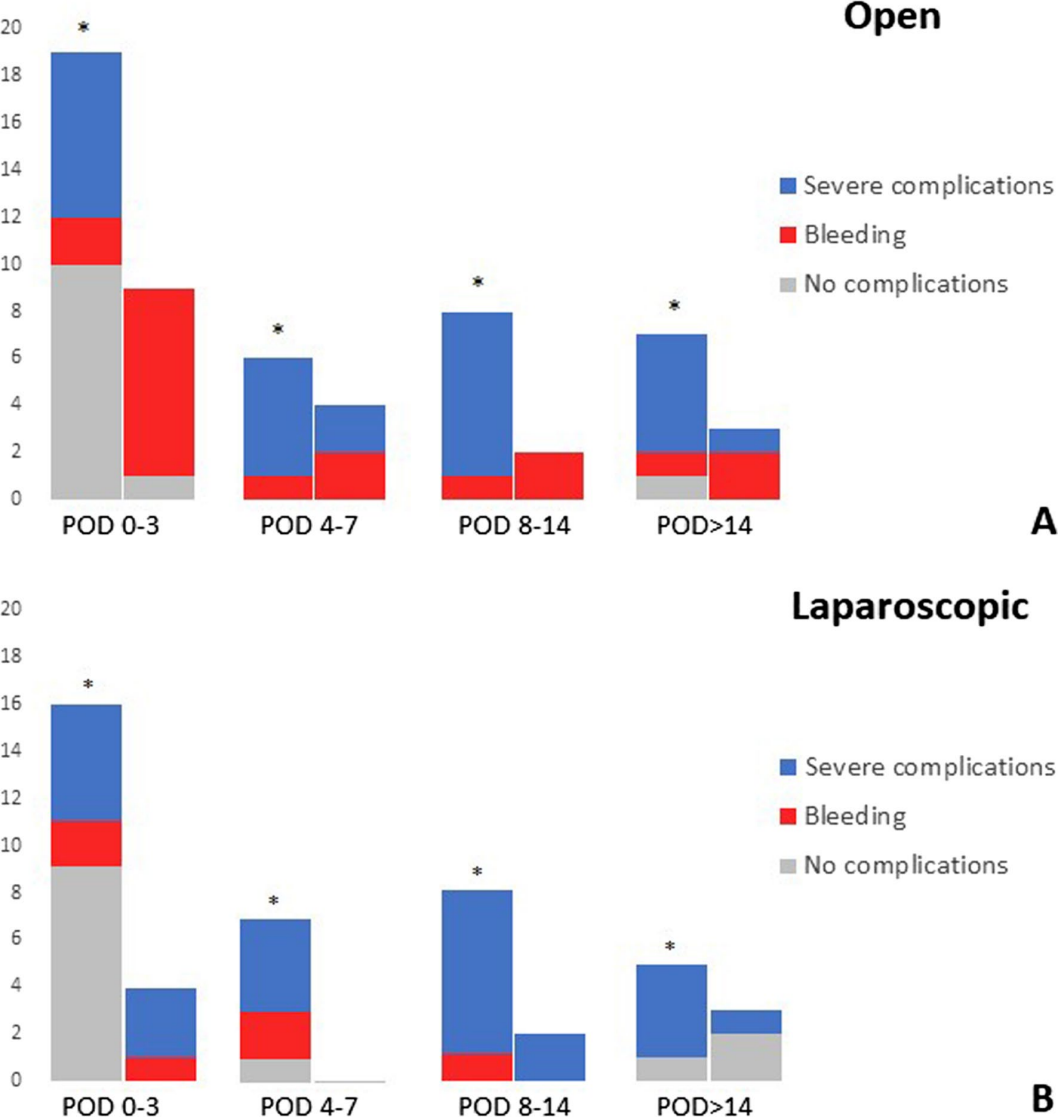
Causes of RBC transfusion for anaemic (*) and non-anaemic patients

Table 3 shows the postoperative transfusion history and the postoperative variation in Hb levels for laparoscopic and open resection cases. The number of patients transfused in the postoperative period was 56 (15.4%) in the open resection group and 47 (12.9%) in the laparoscopic resection group (*P* = 0.395). Similarly, no difference was observed in the total number of RBC units transfused, as well as in the mean ± standard deviation (SD) number of transfused units per patient (*P* = 0.608). Among transfused patients, the mean ± SD number of transfused RBC units (3.7 ± 3.5 versus 3.6 ± 3.4; *P* = 0.828) and the percentage of patients transfused with three or more units (4.7% versus 5.2%; *P* = 0.615) was similar between the two groups. Additional file 1: Fig. S1 reports the timing of postoperative RBC transfusions for the laparoscopic and open resection groups. Patients who received RBC transfusion in the first postoperative days (POD 0–3) where mainly those who presented moderate (*n* = 34) and mild anaemia (*n* = 20). On the other hand, many patients transfused on POD 4–7 (*n* = 13) and the vast majority of those transfused after POD 7 (*n* = 17) presented a severe postoperative complication. Figure 2 illustrates the causes of RBC transfusion according to the presence of preoperative anaemia for laparoscopic and open groups. Patients who presented preoperative anaemia were transfused more frequently, even when no complications occurred. Conversely, in non-anaemic patients, RBC transfusions were required almost entirely in cases with a complicated postoperative course. When no complications occurred, 1 out of 145 (0.7%) and 2 out of 168 (1.2%) in open and laparoscopic group respectively required postoperative RBC transfusions.

**Table 3:** Postoperative Hb and RBC transfusions data for the patients under study according to treatment group

	Open resection *n* = 364	Laparoscopic resection *n* = 364	*P*
Postoperative RBC transfusions	56 (15.4)	47 (12.9)	0.395
Number of postop. RBC units, total	199	174	
Number of postop. RBC units, mean ± SD			
All patients	0.6 ± 1.8	0.5 ± 1.8	0.608
Transfused patients only	3.6 ± 3.4	3.7 ± 3.5	0.828
Number of postop. RBC units			0.615
Two or less	37 (10.2)	30 (8.2)	
Three or more	19 (5.2)	17 (4.7)	
Postop. Hb drop, g/dL, mean ± SD	2.6 ± 1.4	2.4 ± 1.4	0.129
Minimum postop. Hb level, g/dL, mean ± SD	10.8 ± 1.7	10.9 ± 1.7	0.355

Numbers in parentheses are percentages unless specified otherwise

*SD* standard deviation

Looking at variations in Hb levels, laparoscopic and open resections were comparable considering mean ± SD Hb drop (2.6 ± 1.4 versus 2.4 ± 1.4; *P* = 0.129) and the lowest Hb level recorded in the postoperative period (10.8 ± 1.7 versus 10.9 ± 1.7; *P* = 0.355).

### Risk factors for postoperative RBC transfusion

In order to determine the independent predictive factors that were related with the requirement of postoperative RBC transfusions (yes versus no), logistic regression analysis was conducted using a multivariable model. Surgical approach (open versus laparoscopic) plus other 14 factors (age, gender, tumour location, ASA physical status, depth of tumour invasion, presence of nodal metastases, presence and grade of preoperative anaemia, preoperative RBC transfusions, occurrence, severity and type of postoperative complications) were included in the analysis. The types and severity of postoperative complications were included in the multivariate model separately, due to their co-linearity. Table 4 reports the results of univariate and multivariable analysis. The factors associated with the need for postoperative RBCs were the presence of preoperative anaemia (OR 4.4, 95% c.i. 1.5–12.4 for mild anaemia, OR 18.2, 95% c.i. 5.9–56.3 for moderate anaemia; *P* < 0.001) and the occurrence of postoperative complications (OR 5.1, 95% c.i. 2.2–11.5; *P* = 0.025). Considering the type of complications, the highest OR was found in case of anastomotic leakage (OR 38.1, 95% c.i. 9.5–153.4; *P* < 0.001). Open compared to laparoscopic approach was not a statistically significant risk factor for postoperative transfusions (OR 1.2, 95% c.i. 0.8–1.9; *P* = 0.34).

**Table 4:** Univariate and multivariable logistic regression analysing risk factors for postoperative RBC transfusion

	Univariate		Multivariable	
	OR (95% CI)	*P*	OR (95% CI)	*P*
Age ≥ median	2.2 (1.4–3–3)	< 0.001		
Male gender	1.1 (0.7–1.6)	0.76		
Tumour location		0.05		
Right colon	1			
Left colon	0.5 (0.3–0.9)			
Rectum	0.8 (0.5–1.3)			
ASA physical status class ≥ 3	3.2 (1.7–6.2)	< 0.001		
Presence of preoperative anaemia^a^		< 0.001		< 0.001
No	1		1	
Mild	3.3 (1.9–5.6)		4.4 (1.5–12.4)	
Moderate	14.6 (8.3–25.8)		18.2 (5.9–56.3)	
Preoperative RBC transfusions	7.4 (3.5–15.9)	< 0.001		
Depth of tumour invasion ≥ pT3	1.5 (1.0–2.3)	0.04		
Presence of nodal metastases (N+)	1.4 (0.9–2.2)	0.14		
Open surgery	1.2 (0.8–1.9)	0.34		
Postoperative complications	8.3 (5.1–13.6)	< 0.001	5.1 (2.2–11.5)	< 0.001
Severe postoperative complications^b^	16.1 (8.5–30.5)	< 0.001	21.8 (7.5–63.4)	< 0.001
Surgical complications^b^	10.3 (6.5–16.4)	< 0.001	8.9 (3.8–20.8)	< 0.001
Anastomotic leakages^b^	19.7 (8.8–44.0)	< 0.001	38.1 (9.5–153.4)	< 0.001
Bleeding^b^	21.8 (8.4–56.6)	< 0.001	14.6 (2.5–83.3)	0.003
Infective complications^b^	4.1 (2.6–6.6)	< 0.001	7.2 (3.0–17.4)	< 0.001

*OR* odds ratio, *95% CI* 95% confidence interval

^a^Preoperative anaemia resulted a significant risk factor at multivariate analysis also when the type of postoperative complications were included in the model separately

^b^Type of postoperative complications were included in multivariate analysis separately

## Discussion

The main findings of this study are: (i) the minimum postoperative Hb levels and Hb drop were similar between laparoscopic and open group; (ii) no differences were observed in terms of rate and number of postoperative RBC transfusions between the laparoscopic and the open group; (iii) the need for postoperative RBCs were mainly related to the presence of preoperative anaemia and occurrence of postoperative complications; (iv) up to 15% of patients undergoing elective, curative resection, for non-metastatic colorectal tumour were transfused postoperatively with at least one unit of RBCs.

No differences were found between laparoscopic and open resections for CRC in terms of postoperative RBC transfusions and Hb drop as demonstrated by the comparisons between the two matched groups and the univariate and multivariable analysis including other risk factors for RBC transfusions. The fact that laparoscopic surgery is not protective against postoperative RBC transfusions may result unexpected for many surgeons. As shown by our results, the statistically significant advantage in blood loss of laparoscopic over open surgery demonstrated by RCTs does not lead to a consequent clinical advantage in terms of transfusions needs. Although surprising, these results should be considered reliable since the estimated difference in blood loss reported in those studies ranges between 70 and 100 mL [12–16], which may not necessarily prompt blood transfusion. Moreover, the EBL reported in our study for laparoscopic and open surgery is comparable with previously published data [12, 13, 18].

Univariate and multivariable analyses demonstrated that preoperative anaemia and postoperative complications, in particular severe postoperative complications and anastomotic leakage, represent the main risk factors for postoperative transfusions. The graphical representation in Fig. 2 and Additional file 1: Fig. S1 showed a bimodal trend for RBC transfusions, which mostly relates to the presence of preoperative anaemia in the early postoperative phase, and to the occurrence of postoperative complications thereafter.

As demonstrated by other studies, both anaemia and blood transfusions are related to an increased risk of postoperative complications [25, 26]. The increase in postoperative complications, the worsening of long-term results, the rise in medical costs, and the shortage of blood donors lead the medical community to develop several blood management protocols [3, 4, 27, 28]. The recent demonstration that preoperative intravenous iron administration increases Hb and iron level and decreases the need for postoperative blood transfusions pushes forward the optimization of these protocols [4, 5, 29].

All these considerations lead us to emphasize the necessity of a complete preoperative evaluation of all factors influencing anaemia and, in particular, of iron status. In this regard, it has been demonstrated that the correction of anaemia via RBC transfusion is less effective compared to intravenous iron administration, since no stimulation of the hematopoietic system occurs after transfusions, hence providing just a temporary correction of anaemia [30]. Conversely, intravenous iron administration helps in correcting anaemia and in refilling iron stores, that will be essential in the postoperative phase to maintain target Hb level [4, 31, 32]. Considering all these factors, preoperative intravenous iron administration, especially using novel formulations (e.g. ferric carboxymaltose), seems the most cost-effective and quickest way to obtain a significant increment in Hb levels and iron deposits, as it often requires a single administration to achieve clinically relevant increase in Hb level [33, 34].

## Limitations

This paper suffers some limitations. The retrospective nature of the study did not allow us to obtain complete data on other relevant variables that may have influenced the requirements of RBC transfusions. Specifically, it was not possible to retrieve from our retrospective database data regarding the amount of crystalloid given during or after surgery. Also, no data were available on preoperative iron status, to better define the type of anaemia and to compare iron status between the two groups, or data regarding preoperative administration of oral or parenteral iron. However, preoperative Hb values were similar in the two groups, therefore we can safely assume that iron status was also comparable. Second, looking at recent guidelines on blood management, the number of transfusions would have been lower than the one reported in the present study. Nonetheless, the criterion adopted to decide for transfusion did not change during the time of study and it was never influenced by the surgical approach. Since the two groups were balanced according to clinical pathological characteristics, it seems unlikely that this would have made these results different.

## Strengths

The study has also several strengths. To our knowledge, the total number of cases included in this study represent the largest cohort of patients analysing RBC transfusions in subjects undergone surgery for colorectal tumours. This number of cases allowed us to perform an accurate case matching and to analyse a representative number of cases treated by open and laparoscopic surgery. Moreover, this is the sole study that analysed the whole perioperative period considering transfusions received between 30 day before and 90 days after the operation. Finally, it is one of the very few studies that considered the variations in Hb levels, that represent an excellent indicator to evaluate the possible role of laparoscopy in affecting the clinical course in terms of blood loss.

## Conclusions

Laparoscopic surgery itself did not influence postoperative haemoglobin drop and requirements for RBC transfusions after CRC resection. Presence of preoperative anaemia and occurrence of postoperative complications represent the major determinants for postoperative RBC transfusions after open as well as laparoscopic CRC resection.

The treatment of anaemia and iron-deficiency should be considered mandatory in all patients scheduled for elective colorectal surgery in order to treat preoperative anaemia and to limit the occurrence of postoperative complications.

## Supplementary Information

**Additional file 1: Figure S1.** Representation of the number of patients transfused on each postoperative day according to surgical approach.

**Additional file 2: Table S1. **Comparison of standardized mean differences (SMD) for matching variables before and after matching.

## Data Availability

The datasets used and analyzed during the current study are available from the corresponding author upon reasonable request.

## Abbreviations

CRC: Colorectal cancer
RBC: Red blood cells
Hb: Haemoglobin
PSM: Propensity score matching
AJCC: American Joint Committee on Cancer
UICC: Union International Contre Le Cancer
EBL: Estimated intraoperative blood loss
POD: Postoperative days
ASA: American Society of Anaesthesiologists status
c.i.: Confidence intervals
