# Supplementary material for: Laparoscopic surgery does not reduce the need for red blood cell transfusion after resection for colorectal tumour: a propensity score match study on 728 patients
Source: BMC Surg. 2022 Mar 31;22:123. doi: 10.1186/s12893-022-01569-0 (PMC8974035; doi:10.1186/s12893-022-01569-0)

**Figure S1.** Representation of the number of patients transfused on each postoperative day according to surgical approach.


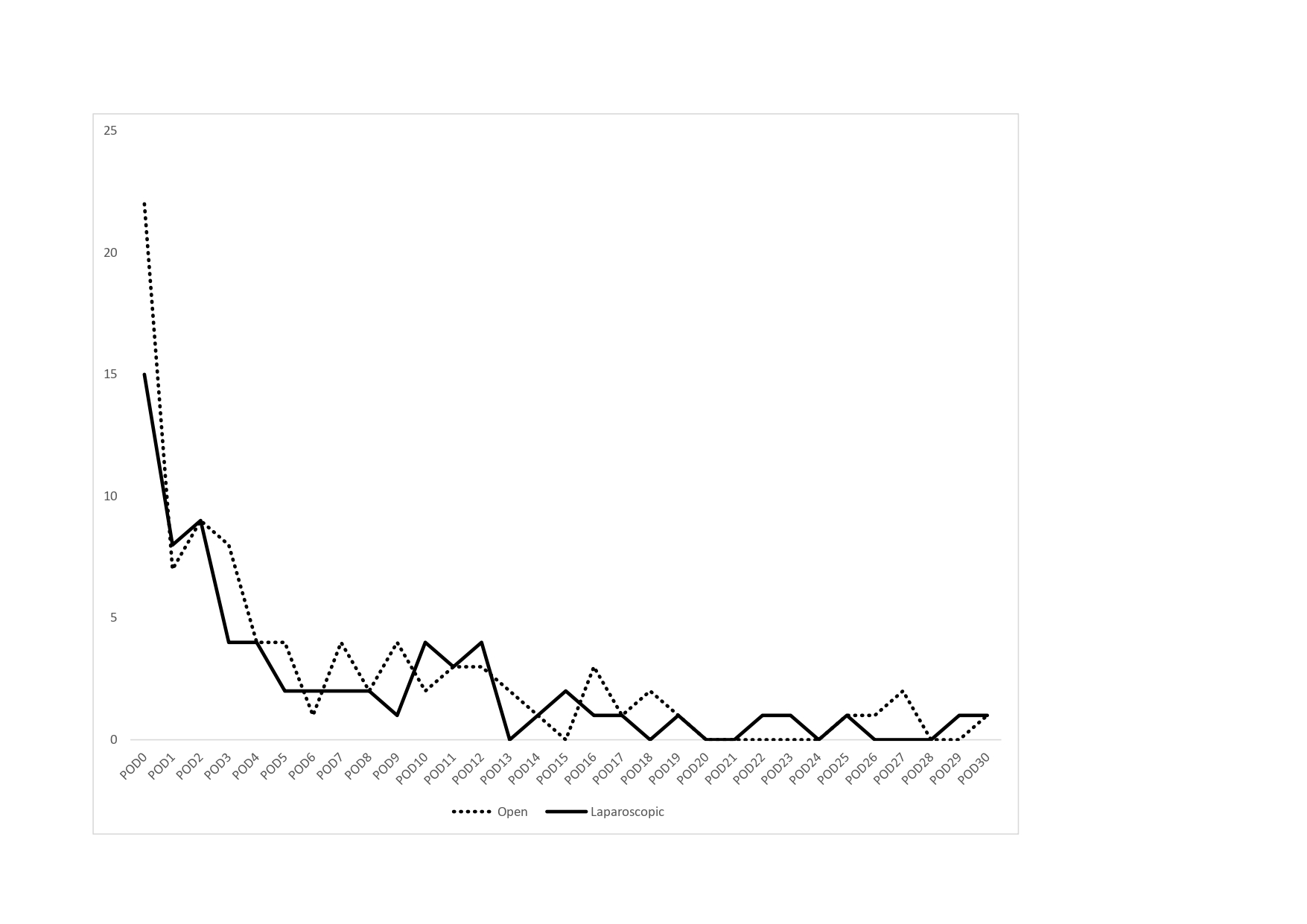

Supplement: Supplementary file 1 — Additional file 1: Figure S1. Representation of the number of patients transfused on each postoperative day according to surgical approach. [file 12893_2022_1569_MOESM1_ESM.docx]
