# Supplementary material for: Laparoscopic surgery does not reduce the need for red blood cell transfusion after resection for colorectal tumour: a propensity score match study on 728 patients
Source: BMC Surg. 2022 Mar 31;22:123. doi: 10.1186/s12893-022-01569-0 (PMC8974035; doi:10.1186/s12893-022-01569-0)
Supplement: Supplementary file 2 — Additional file 2: Table S1. Comparison of standardized mean differences (SMD) for matching variables before and after matching. [file 12893_2022_1569_MOESM2_ESM.docx]

**Table S1.** Comparison of standardized mean differences (SMD) for matching variables before and after matching.

|  | ***Whole cohort*** | | | ***Matched cohort*** | | |
| --- | --- | --- | --- | --- | --- | --- |
|  | ***Open resection***  **(*n* = 770)** | ***Laparoscopic resection***  **(*n* = 401)** | ***SMD*** | ***Open resection***  **(*n* = 364)** | ***Laparoscopic resection***  ***(n* = 364*)*** | ***SMD*** |
| Age*,* years, median (IQR) | 68.8 ± 12.1 | 66.2 ± 11.7 | 0.276 | 66.9 ± 10.6 | 66.5 ± 10.8 | 0.018 |
| Gender, males | 459 (59.6) | 215 (53.6) | 0.118 | 211 (58) | 196 (53.8) | 0.022 |
| UICC R0 | 744 (96.6) | 391 (97.5) | 0.014 | 356 (97.8) | 356 (97.8) | 0.037 |
| Stage TNM 0-I | 241 (31.2) | 190 (47.4) | 0.342 | 171 (46.9) | 180 (49.4) | 0.049 |
| Stage TNM II | 291 (37.8) | 101 (25.2) | 0.307 | 95 (26.1) | 92 (25.3) | 0.013 |
| Stage TNM III | 238 (30.9) | 110 (27.4) | 0.087 | 98 (26.9) | 92 (25.3) | 0.044 |
| No preoperative RBC transfusions | 663 (86.1) | 377 (94) | 0.415 | 350 (96.2) | 349 (95.9) | <0.001 |
| Pre-operative Hb | 12.8 ± 2.1 | 13.2 ± 1.9 | 0.199 | 13.3 ± 1.8 | 13.3 ± 1.8 | 0.045 |

Values in parentheses are percentages unless differently specified.

IQR: interquartile range. SMD: standardized mean difference (absolute values are reported).
